# Longitudinal trajectories of urinary albumin-to-creatinine ratio and risk of proteinuria among Chinese patients with type 2 diabetes: a single−center retrospective cohort study

**DOI:** 10.3389/fendo.2026.1833972

**Published:** 2026-06-02

**Authors:** Qian Chen, Tieqiao Wang, Xiaoqing Tian, Qiankai Jin, Li Li, Yushan Mao, Guoqing Huang

**Affiliations:** 1 Department of Endocrinology, The First Affiliated Hospital of Ningbo University, Ningbo, Zhejiang, China; 2 Department of Medical Record Statistics, The First Affiliated Hospital of Ningbo University, Ningbo, Zhejiang, China; 3 Department of Gastroenterology, Zhenhai District People’s Hospital, Ningbo, China; 4 Department of Endocrinology, Beilun District People’s Hospital, Ningbo, China

**Keywords:** group-based trajectory modeling, proteinuria, retrospective cohort, type 2 diabetes mellitus, urinary albumin-to-creatinine ratio

## Abstract

**Background:**

Urinary albumin-to-creatinine ratio (UACR) is a key marker for monitoring proteinuria progression in type 2 diabetes mellitus (T2DM). However, UACR trajectory patterns and their association with proteinuria risk remain underexplored.

**Methods:**

This retrospective cohort study included 3,101 T2DM patients (baseline UACR <30 mg/g) with regular follow-up from March 2018 to October 2024 at the Ningbo Metabolic Management Center (MMC) subcenter. Clinical data were obtained from electronic medical records. Group-based trajectory modeling (GBTM) identified UACR trajectory patterns. Partial Least Squares Discriminant Analysis (PLS-DA) with Boruta algorithm selected key variables associated with trajectories. Additionally, we used the Light Gradient Boosting Machine (LightGBM) algorithm for multi-class classification modeling and Shapley Additive exPlanations (SHAP) values to quantify individual feature contributions to distinct trajectories. Multivariable Cox regression evaluated proteinuria risk by trajectory.

**Results:**

GBTM analysis identified three distinct UACR trajectories: low-normal, mid-range normal, and rising with fluctuation groups. PLS-DA with Boruta algorithm selected 11 significant features, including baseline UACR, sex, height, Hb, HCT, BMI, waist circumference, RBC, FCP, SCR, and FINS. Meanwhile, we explained the prediction results of a multi-class LightGBM model by assigning SHAP values to 11 features. Multivariable Cox regression showed significantly increased proteinuria risk in both mid-range normal (HR = 48.40, 95% CI: 14.67-159.67) and rising with fluctuation groups (HR = 509.56, 95% CI: 157.01–1653.70) compared to low-normal group.

**Conclusions:**

Identifying distinct UACR trajectories in Chinese T2DM patients, particularly the strong association between rising with fluctuation pattern and proteinuria risk, indicates that trajectory-informed patient classification could enhance early-stage diabetic kidney disease screening and preventive strategies.

## Introduction

Type 2 diabetes mellitus (T2DM) and its complications have caused a heavy global burden on public health. Diabetic kidney disease (DKD), as one of the most significant microvascular complications of T2DM, is the leading cause of end-stage renal disease worldwide, which is associated with high morbidity and mortality (1, 2). It develops in approximately 40% of patients with diabetes, after 10 years of T2DM were diagnosed (3, 4). In China, with rapid economic growth and urbanization, it is estimated that the number of patients with diabetic chronic kidney disease has reached 24.3 million (5). Accordingly, exploring early warning indicators and risk prediction models for DKD holds significant clinical importance for delaying disease progression and improving patient outcomes.

Proteinuria, particularly persistent proteinuria, is a critical clinical marker for the onset and progression of DKD, and its presence indicates progressive decline in renal function (6). The urinary albumin-to-creatinine ratio (UACR), a simple, non-invasive, and reliable test, is widely used for the screening and monitoring of early kidney damage. A single UACR measure has been used to derive risk in most cohort studies (7). Studies have demonstrated that a UACR reduction of 30% per year is associated with a hazard ratio (HR) of 0.7 for the risk of nephropathy outcome (8). However, this conventional single or sporadic UACR measurement have significant limitations—a cross-sectional perspective that fails to capture the dynamic patterns of how an individual’s UACR changes over time (9). Identifying these underlying, heterogeneous longitudinal trajectories is crucial for accurately predicting the risk of proteinuria, gaining deeper insights into the heterogeneity of the disease, and implementing early, personalized interventions.

The emergence of statistical methods such as group-based trajectory modeling (GBTM) cluster-based trajectory models has provided new insights for studying the dynamic changes in UACR. Unlike traditional models that estimate population−average trends, GBTM identifies latent subgroups with distinct patterns, yielding clinically interpretable categorical risk strata that are well suited to medical decision−making. This approach can identify latent subgroups within follow-up populations that exhibit similar change patterns, thereby revealing the heterogeneity of disease progression (10). Reflecting its growing adoption in diabetes research, a recent large−scale study applied model to identify distinct HbA1c trajectories that independently predicted incident cardiovascular disease in T2DM patients (11). Previous studies of clinic-based Chinese people have demonstrated that distinct UACR trajectories correlate closely with major adverse cardiac events, particularly with the long-term elevated trajectory being significantly associated with poor prognosis (12). However, data on the impact of the longitudinal patterns of UACR on the proteinuria risk in patients with T2DM are sparse. Additionally, beyond describing trajectory characteristics, identifying predictors associated with different trajectories is crucial for early identification of high-risk individuals and informing targeted interventions. Currently, systematic research combining multiple statistical learning methods to explore UACR trajectory predictors remains limited.

Based on the above background, this study utilizes longitudinal follow-up data from the Ningbo Metabolic Management Center (MMC) subcenter to investigate the dynamic trajectories of UACR in Chinese patients with T2DM and its association with proteinuria risk. By identifying distinct UACR trajectory subgroups, the study will analyze their relationship with clinical outcomes and explore relevant predictive factors, aiming to provide new evidence-based medical insights for the early prevention and treatment of diabetic nephropathy. The findings are expected to provide crucial reference for establishing risk prediction models tailored to the Chinese population, thereby advancing the implementation of precision prevention and treatment strategies.

## Methods

### Study design and participants

This retrospective cohort study enrolled 6,322 T2DM individuals with regular follow-up from March 2018 to October 2024 at the Ningbo MMC subcenter. T2DM was diagnosed based on the Chinese Diabetes Diagnosis and Treatment Guidelines (13), including fasting blood glucose (FBG) levels of ≥ 7.0 mmol/L, 2-hour blood glucose levels of ≥ 11.1 mmol/L, or a glycated hemoglobin level of ≥ 6.5%. At the time of enrollment into the MMC program, demographic data, relevant complications and biochemical parameters were obtained through questionnaire surveys and laboratory examinations according to a standardized operating procedure. The UACR index was calculated as the urinary albumin (mg)/creatinine (g), and GBTM was used to identify the trajectory of UACR during the exposure period (2018–2024). The baseline exclusion criteria were as follows: (1) patients with age < 18 or age > 75; (2) patients with UACR ≥ 30 mg/g; (3) patients without proteinuria measurement data; (4) patients with proteinuria. The participant retention and follow-up times were shown in **Supplementary Figure 1**. Participants with at least two UACR measurements during follow-up were included. Ultimately, 3101 participants were included in the study.

### Laboratory examination

A trained dedicated MMC nurse uniformly measured waist circumference, systolic blood pressure, and diastolic blood pressure. Waist circumference was measured with the patient wearing light clothing. Blood pressure was measured using an electronic sphygmomanometer after 5 minutes of seated rest. All patients fasted for 10–12 hours prior to biochemical testing. Fasting venous blood samples were collected from the antecubital vein in the morning on the day of examination to measure HbA1C, triglyceride (TG), total cholesterol (TC), and serum creatinine. Urine samples were collected to determine UACR.

Clinical baseline data encompassed the participants’ general characteristics [age, gender, education, annual household income, height, weight, head circumference, neck circumference, hip circumference, waist circumference, systolic blood pressure (SBP), diastolic blood pressure (DBP), and heart rate (HR)], lifestyles (history of smoking and drinking), blood cell counts [hemoglobin (Hb), red blood cell count (RBC), white blood cell count (WBC), platelet count (PLT), Hematocrit (HCT), mean red blood cell volume (MCV), mean corpuscular hemoglobin (MCH), and mean platelet volume (MPV)], biochemical indicators [fasting plasma glucose (FPG), 2-hour postprandial plasma glucose (2hPG), fasting insulin (FINS), 2-hour postprandial insulin (2hPI), fasting C-peptide (FCP), 2-hour postprandial C-peptide (2hCP), alanine aminotransferase (ALT), aspartate aminotransferase (AST), Alkaline Phosphatase (ALP), gamma-glutamyl transpeptidase (GGT), albumin (ALB), Blood Urea Nitrogen (BUN), Serum Creatinine (SCR), uric acid (UA), triglycerides (TG), total cholesterol (TC), high-density lipoprotein cholesterol (HDL-C), and low-density lipoprotein cholesterol (LDL-C)], and other laboratory value [glycosylated hemoglobin (HbA1c)].

### Proteinuria

Proteinuria is diagnosed by quantifying 24-hour urinary protein excretion (14). For early detection, a spot urine albumin-to-creatinine ratio (UACR) of 30–300 mg/g defines microalbuminuria, while a UACR >300 mg/g indicates clinical (overt) albuminuria.

### Statistical analysis

We employed the Kolmogorov-Smirnov (K-S) test to assess the normality of continuous variables. The mean ± standard deviation was used to describe continuous variables with normal distribution, and one-way ANOVA was used for inter-group comparisons. The median (Q1–Q3) was used to represent the continuous variables with a non-normal distribution, and Wilcoxon’s rank-sum test was applied for inter-group comparison. Case numbers and percentages performed qualitative data, and the chi-square test was applied for inter-group comparisons. And the standardized mean difference (SMD) was used to evaluate balance between groups (15). We employed multiple imputation to handle missing data in the baseline characteristics, as the missingness for any variable was below 20% (**Supplementary Figure 2**). The cumulative hazard of proteinuria was estimated utilizing Kaplan–Meier curves, with estimates of the difference between the curves by log rank.

GBTM is a special case of latent class growth curves where individuals within the same group are assumed to follow the same trajectory (16). Its key outputs are the trajectory shape (typically parameterized as a polynomial function of time) and the posterior probability of trajectory group membership (17). In this study, GBTM was implemented through R to identify distinct trajectory patterns of UACR. Firstly, based on the principle that the proportion of each group must be over 5% of the total participants, the models with 3, 2, and 1 trajectory patterns were all fitted. Then, by comparing the Bayesian Information Criterion (BIC) and Akaike Information Criterion (AIC) of distinct models, we identified the most appropriate model with 3 patterns (**Supplementary Table 1**).

Given the high dimensionality, strong collinearity, and multiclass outcome of our dataset, traditional variable selection is limited by instability and an inability to capture non-linear relationships. We therefore employed a complementary two−step strategy: first, Partial Least Squares Discriminant Analysis (PLS−DA) was used for supervised dimensionality reduction, projecting predictors onto latent components that maximize covariance with the three trajectory classes to isolate variables with the greatest discriminatory power; second, the Boruta algorithm, a Random Forest wrapper, was applied to identify all relevant features by comparing each variable’s importance against randomly permuted shadow attributes, capturing individually important predictors that PLS−DA may overlook. Agreement between the two methods constitutes a conservative triangulation that reduces false positives.

To further interpret the classification logic underlying UACR trajectory categorization, we deployed the Light Gradient Boosting Machine (LightGBM) algorithm, a gradient boosting framework that flexibly models non−linear and interactive effects without the constraints of conventional regression. The input features were those identified through the PLS−DA with Boruta pipeline. SHapley Additive exPlanations (SHAP) values were then used to decompose predictions into additive feature contributions, yielding both global importance and patient−level explanations (18).

The estimation of hazard ratios (HRs), to evaluate proteinuria risk, was accomplished utilizing the Cox proportional hazards regression models. The proportional hazard assumption was assessed with Schoenfeld residuals, with no variables violated. Multivariable models were conducted after adjustment for age, gender, annual household income, systolic blood pressure, height, waist circumference, fasting plasm glucose, HbA1c, white blood cell, blood urea nitrogen, total cholesterol, HDL, LDL, and baseline UACR.

Statistical analyzes were conducted using the R language (version 4.2.3, http://www.R-project.org/). GBTM was conducted using the “lcmm” package; PLS-DA with Boruta algorithm was performed using the “ropls” and “Boruta” packages; LightGBM was implemented using the “lightgbm” package, with SHAP values calculated via “shapviz” package; and multivariable Cox regression was performed using the “survival” package. All data were analyzed using two-sided tests, and statistical significance was defined as *P* < 0.05.

## Results

### Characteristics of participants by UACR trajectories

**Figure 1:**
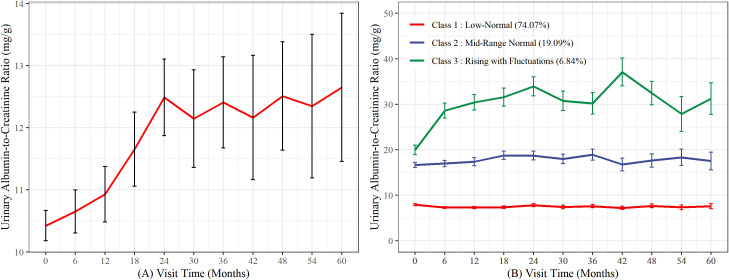
The UACR trajectory during 2018–2024. **(A)** The trajectory of UACR changes in the overall participants during 2018–2024. **(B)** Three UACR trajectory based on BGTM during 2018–2024.

Baseline characteristics of subjects was presented in **Table 1**. At baseline, we included 3,101 T2DM individuals from 2018 to 2024, who had median age of 52.00 (IQR: 43.00-59.00). Among the participants, 2,011 (64.85%) were male and 1090 (35.15%) were female. GBTM analysis identified three distinct trajectories based on the UACR change patterns from 2018 to 2024, as depicted in **Figure 1**. These patterns included low-normal group (n = 2,297, median age = 51.00 (42.00, 58.00), median UACR = 6.87 (4.70, 10.25)), mid-range normal group (n = 592, median age = 55.00 (45.75, 61.00), median UACR = 16.77 (12.04, 21.69)), and rising with fluctuation group (n = 212, median age = 56.00 (47.00, 62.00), median UACR = 21.40 (14.30, 26.74)). These trajectories showed significant baseline differences in gender, age, annual household income, systolic blood pressure, height, BMI, waist circumference, fasting plasm glucose, HbA1c, white blood cell, blood urea nitrogen, total cholesterol, HDL, LDL, and baseline UACR (all P < 0.05, SMD > 0.1).

**Table 1:** Baseline characteristics of subjects according to the trajectories of UACR from 2018 to 2024.

Characteristic	Total	Low-normal	Mid-range normal	Rising with fluctuation	P	SMD
	3101	2297	592	212		
Age	52.00 (43.00, 59.00)	51.00 (42.00, 58.00)	55.00 (45.75, 61.00)	56.00 (47.00, 62.00)	<0.001	0.179
Gender (Male), %	2011 (64.85)	1540 (67.04)	338 (57.09)	133 (62.74)	<0.001	0.137
Education (High school or above), %	1631 (52.60)	1244 (54.16)	282 (47.64)	105 (49.53)	0.012	0.087
Annual household income (≥100,000), %	2008 (64.75)	1519 (66.13)	367 (61.99)	122 (57.55)	0.013	0.118
Smoking (Yes), %	939 (30.28)	716 (31.17)	160 (27.03)	63 (29.72)	0.145	0.061
Drinking (Yes), %	1583 (51.05)	1197 (52.11)	283 (47.80)	103 (48.58)	0.132	0.057
SBP	131.37 ± 17.02	130.27 ± 16.61	133.89 ± 17.40	136.14 ± 18.74	<0.001	0.223
DBP	78.00 (72.00, 86.00)	78.00 (71.00, 85.00)	79.00 (72.00, 86.00)	80.00 (71.00, 86.25)	0.3	0.061
HR	81.00 (74.00, 90.00)	81.00 (74.00, 90.00)	82.00 (74.00, 91.25)	81.00 (73.75, 90.00)	0.095	0.076
Height	166.00 (159.00, 171.50)	166.50 (159.50, 172.00)	164.50 (158.00, 170.50)	164.00 (158.00, 170.62)	<0.001	0.130
Weight	68.80 (61.00, 77.30)	68.90 (61.10, 77.10)	69.00 (60.90, 79.00)	68.50 (60.10, 76.93)	0.486	0.073
BMI	25.10 (22.90, 27.50)	25.00 (22.80, 27.30)	25.50 (23.30, 28.10)	25.20 (22.98, 27.33)	0.002	0.108
Head circumference	56.00 (54.50, 57.50)	56.00 (54.50, 57.50)	56.00 (54.00, 58.00)	56.00 (54.00, 57.00)	0.335	0.117
Neck circumference	37.00 (35.00, 40.00)	37.00 (35.00, 40.00)	37.00 (35.00, 40.00)	37.00 (35.00, 39.00)	0.694	0.047
Hip circumference	95.00 (91.00, 99.00)	95.00 (91.00, 99.50)	95.00 (91.00, 100.00)	94.00 (90.00, 98.00)	0.146	0.126
Waist circumference	89.40 ± 10.31	89.00 ± 10.29	90.73 ± 10.72	90.02 ± 8.99	0.001	0.114
FPG	7.65 (6.49, 9.62)	7.59 (6.44, 9.49)	7.88 (6.71, 9.79)	8.00 (6.65, 10.71)	0.006	0.106
2hPG	13.85 ± 5.10	13.80 ± 5.11	13.79 ± 4.83	14.59 ± 5.71	0.091	0.100
FINS	54.28 (29.31, 90.35)	53.08 (28.83, 87.64)	56.57 (31.08, 98.10)	57.04 (30.37, 92.65)	0.108	0.071
2hPI	186.70 (92.58, 326.70)	186.40 (92.54, 328.20)	189.95 (95.54, 324.34)	189.90 (91.00, 303.85)	0.941	0.049
FCP	0.76 (0.55, 1.07)	0.75 (0.55, 1.04)	0.81 (0.57, 1.16)	0.80 (0.57, 1.12)	0.002	0.092
2hCP	1.87 (1.31, 2.76)	1.87 (1.32, 2.72)	1.87 (1.28, 2.80)	1.90 (1.32, 2.89)	0.913	0.022
HbA1c	7.40 (6.50, 9.10)	7.30 (6.40, 8.90)	7.70 (6.60, 9.20)	7.75 (6.77, 9.60)	0.001	0.126
Hb	148.00 (137.00, 158.00)	148.00 (137.00, 158.00)	145.00 (135.00, 157.00)	148.00 (136.00, 157.25)	0.008	0.089
RBC	4.90 (4.54, 5.27)	4.90 (4.55, 5.27)	4.90 (4.50, 5.23)	4.92 (4.54, 5.35)	0.225	0.061
WBC	6.19 (5.15, 7.35)	6.11 (5.13, 7.29)	6.30 (5.20, 7.50)	6.30 (5.20, 7.66)	0.049	0.113
PLT	227.43 ± 60.90	227.51 ± 60.75	228.47 ± 60.03	223.66 ± 64.99	0.61	0.051
HCT	43.99 ± 4.37	44.12 ± 4.25	43.48 ± 4.67	43.97 ± 4.70	0.007	0.093
MCV	90.00 (87.20, 92.90)	90.00 (87.40, 93.00)	89.95 (87.00, 92.30)	89.50 (86.88, 92.20)	0.055	0.066
MCH	30.10 (29.10, 31.10)	30.10 (29.20, 31.20)	30.00 (29.00, 31.00)	30.00 (29.00, 30.90)	0.034	0.047
MPV	10.30 (9.70, 10.90)	10.20 (9.70, 10.90)	10.30 (9.70, 10.90)	10.40 (9.70, 11.00)	0.335	0.051
ALT	25.00 (18.00, 39.00)	25.00 (18.00, 39.00)	25.00 (18.00, 38.00)	24.00 (18.00, 37.00)	0.769	0.044
AST	22.00 (18.00, 29.00)	22.00 (18.00, 28.00)	22.00 (18.00, 29.00)	22.00 (17.75, 29.00)	0.394	0.002
ALP	80.00 (66.00, 96.00)	79.00 (65.00, 96.00)	82.00 (67.00, 98.00)	79.00 (67.75, 97.00)	0.085	0.064
GGT	27.00 (19.00, 45.00)	27.00 (18.00, 45.00)	27.00 (19.00, 44.00)	26.00 (19.00, 48.00)	0.802	0.036
ALB	44.80 (42.40, 46.80)	44.80 (42.50, 46.80)	44.70 (42.40, 46.80)	44.60 (42.18, 46.52)	0.471	0.073
BUN	5.12 (4.32, 6.04)	5.11 (4.32, 6.02)	5.15 (4.27, 6.05)	5.28 (4.40, 6.19)	0.22	0.115
SCR	63.00 (53.00, 74.00)	64.00 (54.00, 74.00)	60.00 (49.00, 72.00)	65.00 (53.00, 75.25)	<0.001	0.108
UA	328.80 (274.70, 390.60)	330.00 (275.70, 390.50)	324.35 (272.17, 386.00)	319.15 (270.03, 397.78)	0.684	0.024
TG	1.39 (0.99, 2.05)	1.37 (0.96, 2.00)	1.49 (1.07, 2.15)	1.48 (1.05, 2.08)	0.002	0.044
TC	4.81 ± 1.23	4.84 ± 1.23	4.80 ± 1.24	4.56 ± 1.24	0.007	0.149
HDL	1.17 (1.01, 1.37)	1.18 (1.01, 1.38)	1.15 (0.99, 1.34)	1.10 (0.96, 1.31)	<0.001	0.168
LDL	3.12 ± 0.92	3.13 ± 0.92	3.12 ± 0.91	2.93 ± 0.93	0.007	0.149
UACR	8.36 (5.29, 14.18)	6.87 (4.70, 10.25)	16.77 (12.04, 21.69)	21.40 (14.30, 26.74)	<0.001	1.269

### PLS-DA with Boruta

**Figure 2:**
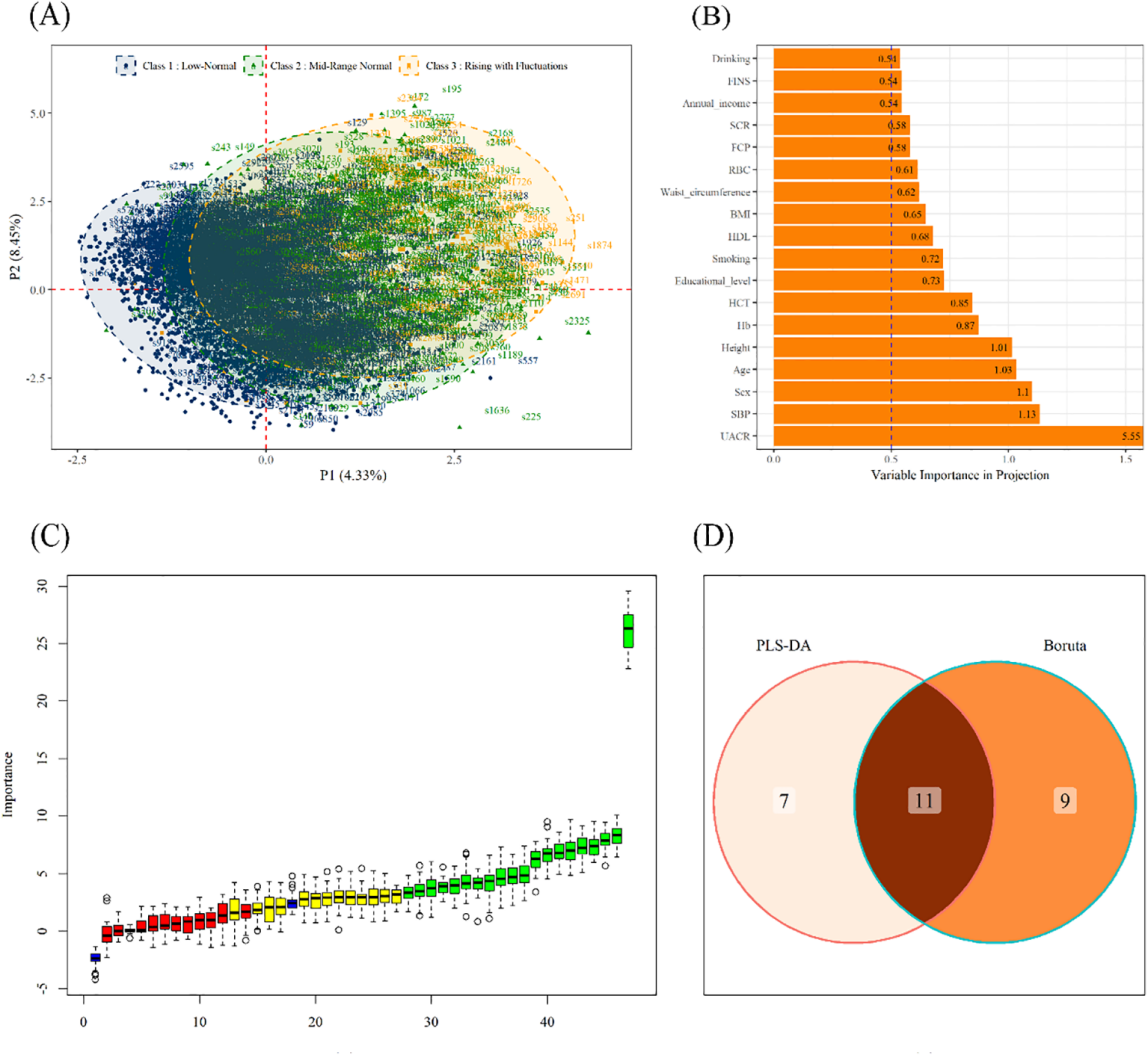
Screening of key predictors based on PLS-DA with Boruta. **(A)** PLS-DA score plot for three trajectories. **(B)** Variable importance in projection (VIP) ranking of predictors based on PLS-DA. **(C)** Feature variables screening based on Boruta. Green markers denoted confirmed important variables, yellow indicated tentative importance, and red represented unimportant variables. **(D)** Venn diagram of PLS-DA with Boruta.

The integration of PLS-DA with Boruta algorithm identified key variables associated with the trajectories of the studied outcomes, as shown in **Figure 2**. The PLS-DA score plot (**Figure 2A**) displayed the separation of samples into three trajectory patterns along the first two principal components, further validating our three-trajectory classification. The Variable Importance in Projection (VIP) plot (**Figure 2B**) ranked the contribution of variables to the PLS-DA model, with baseline UACR showing the highest VIP value, followed by SBP, sex, age, height, and other variables, indicating their strong predictive power for trajectory classification. **Figure 2C** quantifies the importance of each variable using the Boruta algorithm. **Figure 2D** illustrates the overlap between variables selected by PLS-DA and Boruta: 11 significant feature variables were identified as key by both methods, including baseline UACR, sex, height, Hb, HCT, BMI, waist circumference, RBC, FCP, SCR, and FINS, 7 variables were identified only by PLS-DA, and 9 variables were identified only by Boruta.

### Prediction of UACR trajectories based on LightGBM

**Figure 3:**
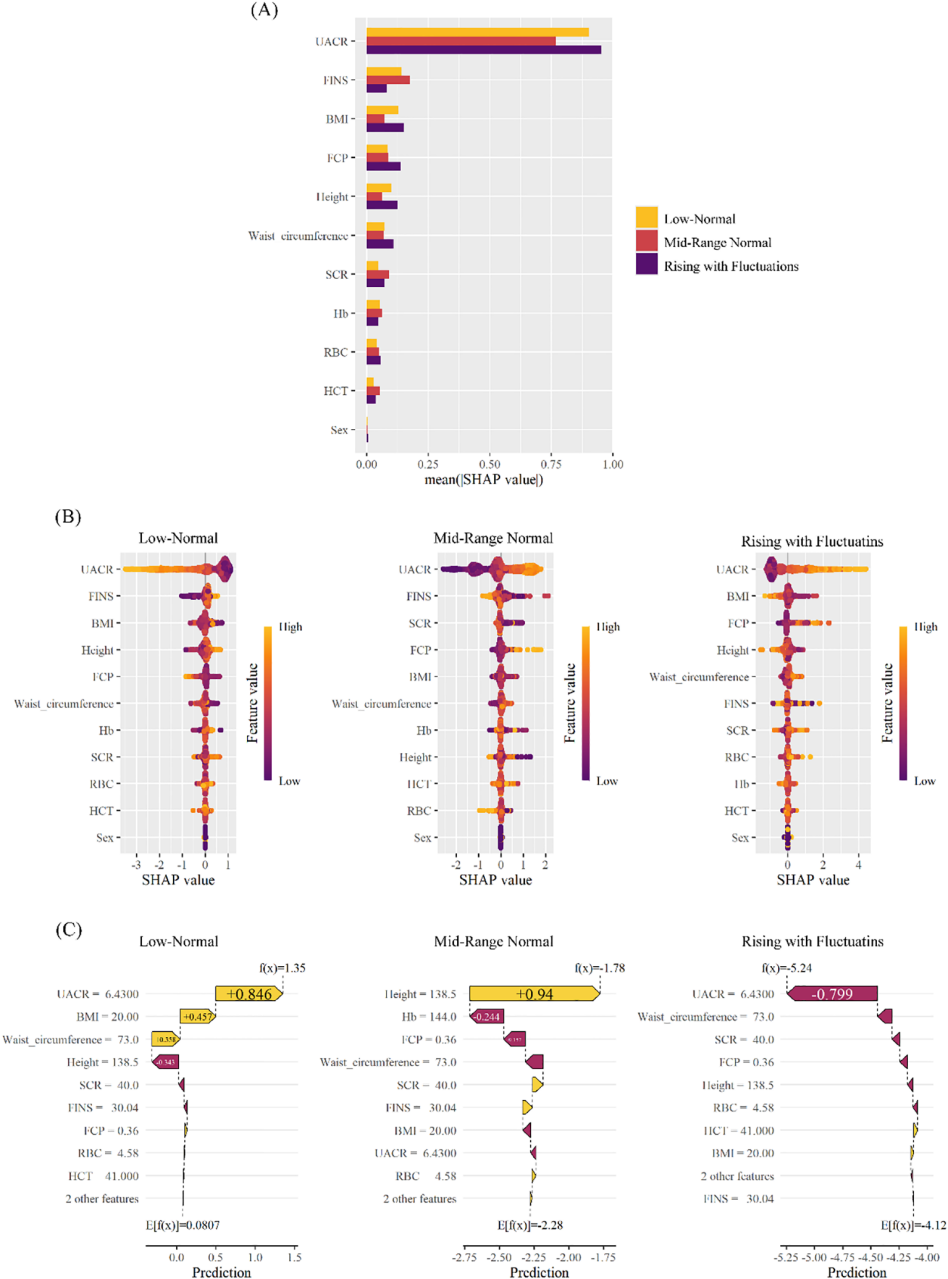
Prediction of UACR trajectories based on LightGBM by assigning SHAP values to 11 key features. **(A)** Mean absolute SHAP values for 11 key features among different UACR trajectories. **(B)**The effect of 11 key features explained by SHAP values among different UACR trajectories. **(C)** Prediction of UACR trajectory patterns for a representative sample based on SHAP values of 11 features.

As shown in **Figure 3**, this plot quantifies the predictive importance of 11 key feature using the mean absolute SHAP value—a metric reflecting the average magnitude of a feature’s contribution to model predictions across all samples. It is evident from **Figure 3A** that the baseline UACR exhibits a substantially higher mean absolute SHAP value than other features, confirming it as the dominant predictive feature for distinguishing UACR trajectory classes. In contrast, features such as sex display near-negligible mean SHAP values, indicating their minimal discriminative value for trajectory classification.

As illustrated in **Figure 3B**, this plot further explores class-specific feature impact patterns, delineating the direction and magnitude of each feature’s effect within individual UACR trajectory classes. In the low-normal trajectory, UACR were strongly associated with positive SHAP values (consistently promoting model predictions for this pattern); In the rising with fluctuation trajectory, UACR values correlated with negative SHAP values (inhibiting predictions for this pattern). For secondary features, pattern-specific SHAP value distributions revealed divergent effects: height, for instance, exhibited opposing directional impacts across low-normal and rising with fluctuations group.

Finally, we concretize these patterns by illustrating feature contributions for a representative sample in each group, showing how individual feature values combine to generate the model’s prediction, as shown in **Figure 3C**. For the low-normal pattern, UACR (6.4300) and BMI (20.00) contributed positively (+0.846, +0.457) to the predicted value (f(x)=1.35), while height (138.5) exerted a negative contribution (-0.343). For the mid-range normal pattern height (138.5) contributed positively (+0.94) to the predicted value (f(x)=-1.78), while Hb (144.0) exerted a negative contribution (-0.244). And for rising with fluctuation pattern, UACR (6.4300) exerted a negative contribution (-0.799) to the predicted value [f(x)=-5.24]. Consequently, we can infer that this individual’s UACR was most likely to exhibit the low-normal pattern.

### Proteinuria risk based on UACR trajectories

**Figure 4:**
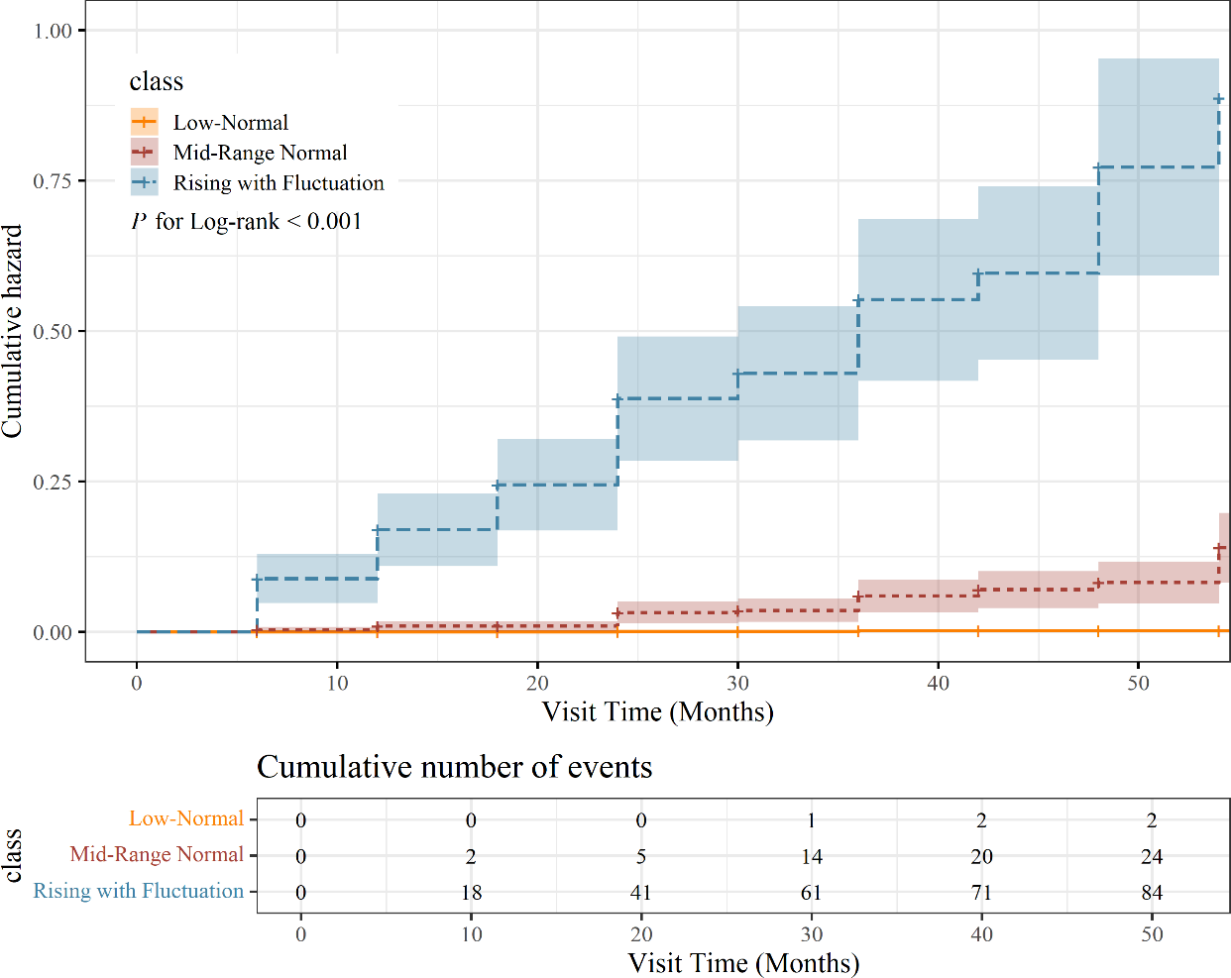
Kaplan–Meier curves of incident proteinuria according to different trajectory groups during 2018–2024.

The Kaplan–Meier curve demonstrated that individuals in the rising with fluctuation group of UACR exhibited a significantly elevated risk of proteinuria compared to other trajectory groups (log-rank test, *P* < 0.001) (**Figure 4**). After adjusting for all covariates, mid-normal group (HR: 39.69, 95%CI: 12.19-129.22) and rising with fluctuation group (HR: 377.35, 95%CI: 119.85-1188.05) were positively related to proteinuria risk, compared with low-normal group (**Table 2**). After adjusting for confounders, multivariable Cox regression showed significantly increased proteinuria risk in both the mid-range normal (HR = 48.40, 95% CI: 14.67-159.67) and rising with fluctuation groups (HR = 509.56, 95% CI: 157.01–1653.70) compared to low-normal group.

**Table 2:** Hazard ratios (HRs) and 95% confidence intervals (95% CIs) of proteinuria risk by UACR trajectory groups.

Group	HR (95% CI)		
	Model 1	Model 2	Model 3
Low-Normal	1.0 (Reference)	1.0 (Reference)	1.0 (Reference)
Mid-Range Normal	39.69 (12.19, 129.22)	39.40 (12.08, 128.49)	48.40 (14.67, 159.67)
Rising with Fluctuation	377.35 (119.85, 1188.05)	379.64 (120.03,1200.79)	509.56 (157.01, 1653.70)

Model 1 was non‐adjusted model; Model 2 was adjusted for age and gender; Model 3 was further adjusted for education, annual household income, SBP, HR, height, head circumference, X2hPG, X2hCP, HbA1c, PLT, ALB, BUN, SCR, TC, HDL, LDL, and baseline UACR.

## Discussion

In our study, a prospective analysis of 3,101 participants with T2DM at the Ningbo MMC subcenter, identified three distinct patterns of UACR trajectories between 2018 and 2024: low-normal, mid-range normal and rising with fluctuation groups. Meanwhile, we employed a dual methodology of PLS-DA and Boruta algorithm to identify 11 significant feature variables associated with these trajectories, including baseline UACR, sex, height, Hb, HCT, BMI, waist circumference, RBC, FCP, SCR, and FINS. These variables have all been shown to be related to changes of UACR in previous studies (19–22). Meanwhile, we explained the prediction results of a multi-class LightGBM model by assigning SHAP values to 11 features. Multivariable Cox regression analysis, after adjusting for potential confounders, revealed that compared with the low-normal group, both the mid-range normal (HR = 48.40, 95% CI: 14.67-159.67) and rising with fluctuation (HR = 509.56, 95% CI: 157.01–1653.70) groups had significantly increased risk of incident proteinuria. These findings highlight the critical role of longitudinal UACR dynamics in predicting proteinuria risk in Chinese T2DM patients, further underscoring the significant value of monitoring UACR dynamics for prognostic assessment.

Most patients with T2DM are the potential population at risk of DKD. Several plausible mechanisms may underpin the observed associations between UACR trajectories and proteinuria risk. First, the rising with fluctuation trajectory likely reflects intermittent hyperglycemia and inadequate glycemic control, which induce oxidative stress and endoplasmic reticulum stress in renal glomerular endothelial cells and podocytes (23–25). These processes impair the integrity of the glomerular filtration barrier, leading to increased albumin leakage and progressive renal damage (26). Second, sustained moderate UACR in the mid-range normal group may indicate subclinical glomerular injury, characterized by early podocyte loss and glomerular basement membrane thickening—pathological changes that precede overt proteinuria (27, 28). Additionally, the key variables identified (e.g., baseline UACR, sex, height) suggest that UACR trajectories are shaped by a combination of renal structural factors, hormonal influences, and metabolic status. For example, sex differences may reflect estrogen-mediated protection of glomerular function in women, while height correlates with nephron number, a key determinant of renal reserve capacity in the context of diabetic metabolic stress (29–31). Collectively, these mechanisms underscore that UACR trajectories are not merely markers of renal injury but reflect underlying pathological processes driving DKD progression.

From a clinical perspective, these trajectory−based classifications directly complement and refine current guideline−based risk assessment, which predominantly relies on single−time−point UACR thresholds. Our data demonstrate that reliance on a single baseline UACR measurement would misclassify a substantial proportion of patients: individuals assigned to the mid−range normal trajectory—who would conventionally be deemed “low risk” based on normoalbuminuria at a single visit—nonetheless carry sustained subclinical risk and warrant structured surveillance (32). Conversely, patients with intermittently normal UACR values but an overall rising−with−fluctuation pattern may not reach treatment−triggering thresholds under current care models, despite harboring the worst renal prognosis. Longitudinal UACR monitoring is readily implementable in routine practice, as electronic health records facilitate the integration of multi−time−point data, allowing clinicians to identify high−risk patients by aligning individual UACR trends with the trajectory templates derived in this study. Such trajectory−informed stratification enables differential management calibrated to projected risk rather than to threshold exceedance alone. Patients in the rising−with−fluctuation group should be prioritized for intensified surveillance (e.g., quarterly UACR and eGFR assessments) and proactive initiation of renin−angiotensin−aldosterone system (RAAS) inhibitors, combined with tighter glycemic control (HbA1c < 7.0%) and lifestyle modifications including sodium restriction and weight management (33–37). Patients in the mid−range normal group, who would be regarded as “low risk” under current single−measurement paradigms, should undergo regular semi−annual surveillance to permit early detection of trajectory worsening before overt proteinuria develops. Embedding UACR trajectory analysis into routine T2DM care thus refines the timing and intensity of intervention—shifting clinical practice from a reactive model triggered by threshold exceedance toward a proactive, trajectory−guided model of early risk modification.

Integrating UACR trajectory analysis into routine T2DM care can enhance the precision of early DKD screening and reduce the burden of ESRD, which aligns with the broader goal of personalized medicine in chronic metabolic diseases (38). Furthermore, the dual methodology combining PLS-DA and the Boruta algorithm serves to robustly identify and validate key clinical features associated with different UACR trajectories, thereby strengthening the biological plausibility and clinical interpretability of the risk stratification. As a result, the final risk classification model based LightGBM becomes more parsimonious, stable, and readily translatable into clinical practice. Together, this data-driven strategy provides methodological assurance for extracting reliable risk trajectories from longitudinal data, thereby supporting clinical decision systems in achieving earlier and more accurate dynamic risk prediction.

This study has several limitations. First, as a retrospective single-center study, the generalizability of the findings to other Chinese T2DM populations or ethnic groups may be limited. And the retrospective design may introduce potential selection bias (e.g., differential inclusion based on data availability) and measurement frequency bias (e.g., non-standardized testing intervals affecting event detection). It will be imperative to conduct studies with expanded sample sizes and extended follow-up periods in future research. Second, we used GBTM analysis to identify three distinct UACR trajectories: low-normal, mid-range normal, and rising with fluctuation groups. However, in the clinic, some patients (<10%) have shown a reduction in UACR and a decreased risk of proteinuria. In this study, we did not discover this subgroup owing to the small size. Third, the limited number of UACR measurements monitored in some participants during follow-up may have led to misinterpretation of trajectory changes and has affected the final results. High-quality prospective studies with expanded sample sizes are urgently needed to validate the findings of this study. In addition, despite accounting for numerous significant confounding factors, we cannot rule out residual confounding effects arising from unadjusted known factors (e.g., use of RAAS inhibitors or SGLT2 inhibitors, duration of diabetes, and comorbidities) as well as unknown or unmeasured confounders (e.g., medication adherence, environmental factors). Future prospective, multicenter studies are needed to validate these trajectories and to investigate the underlying biological mechanisms driving different UACR progression patterns.

## Conclusion

This study identified distinct UACR trajectories among Chinese T2DM patients, particularly highlighting the strong association between rising with fluctuation patterns and proteinuria risk. Our findings support the use of trajectory-based patient stratification to improve early DKD screening and optimize preventive strategies. In the future, large-scale, high-quality studies are warranted to validate the predictive value of the longitudinal UACR trajectories for in T2DM patients.

## Data Availability

The original contributions presented in the study are included in the article/**Supplementary Material**. Further inquiries can be directed to the corresponding authors.
