# Supplementary material for: Longitudinal trajectories of urinary albumin-to-creatinine ratio and risk of proteinuria among Chinese patients with type 2 diabetes: a single−center retrospective cohort study
Source: Front Endocrinol (Lausanne). 2026 Jun 2;17:1833972. doi: 10.3389/fendo.2026.1833972 (PMC13268924; doi:10.3389/fendo.2026.1833972)
Supplement: Supplementary file 1 [file DataSheet1.docx]

**
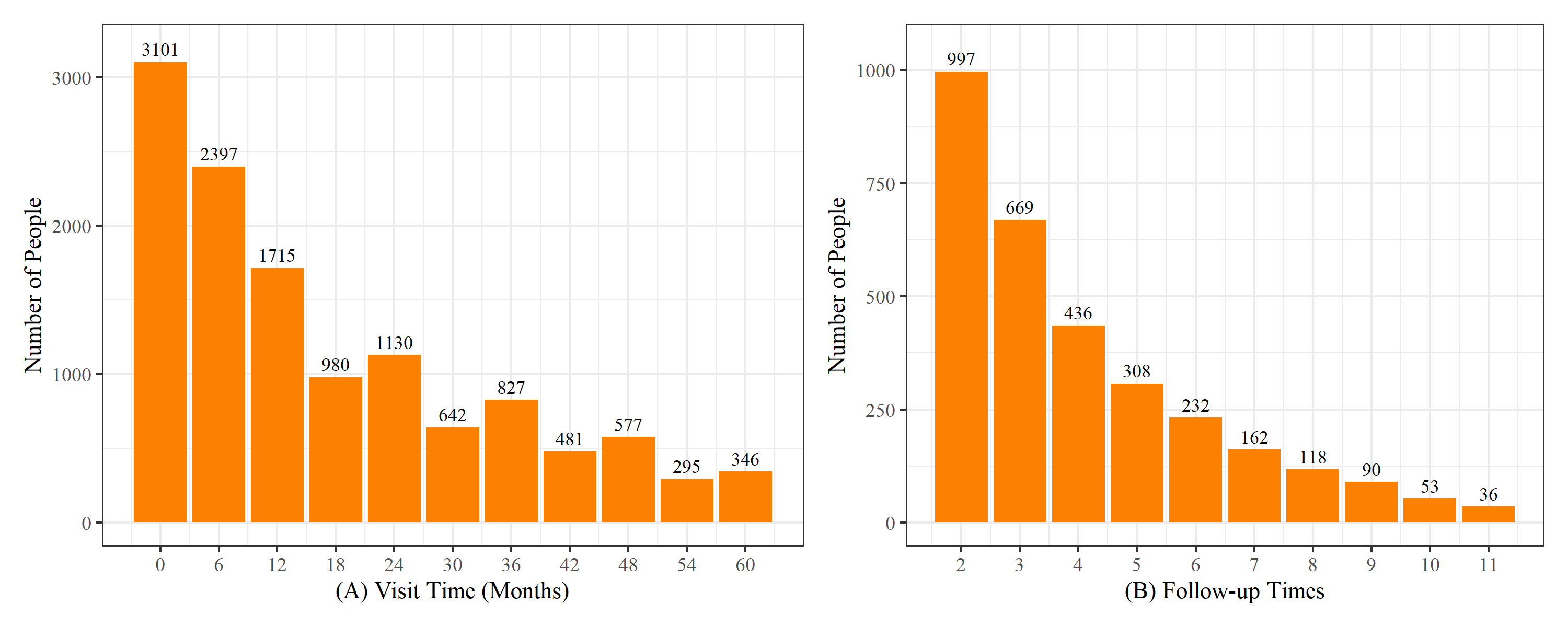
** **Supplementary Figure 1. Participant retention and follow-up times** **from 2018 to 2024.**

(A) Number of participants at different visit time (every 6 months). (B) Distribution of participants by total follow‑up times.


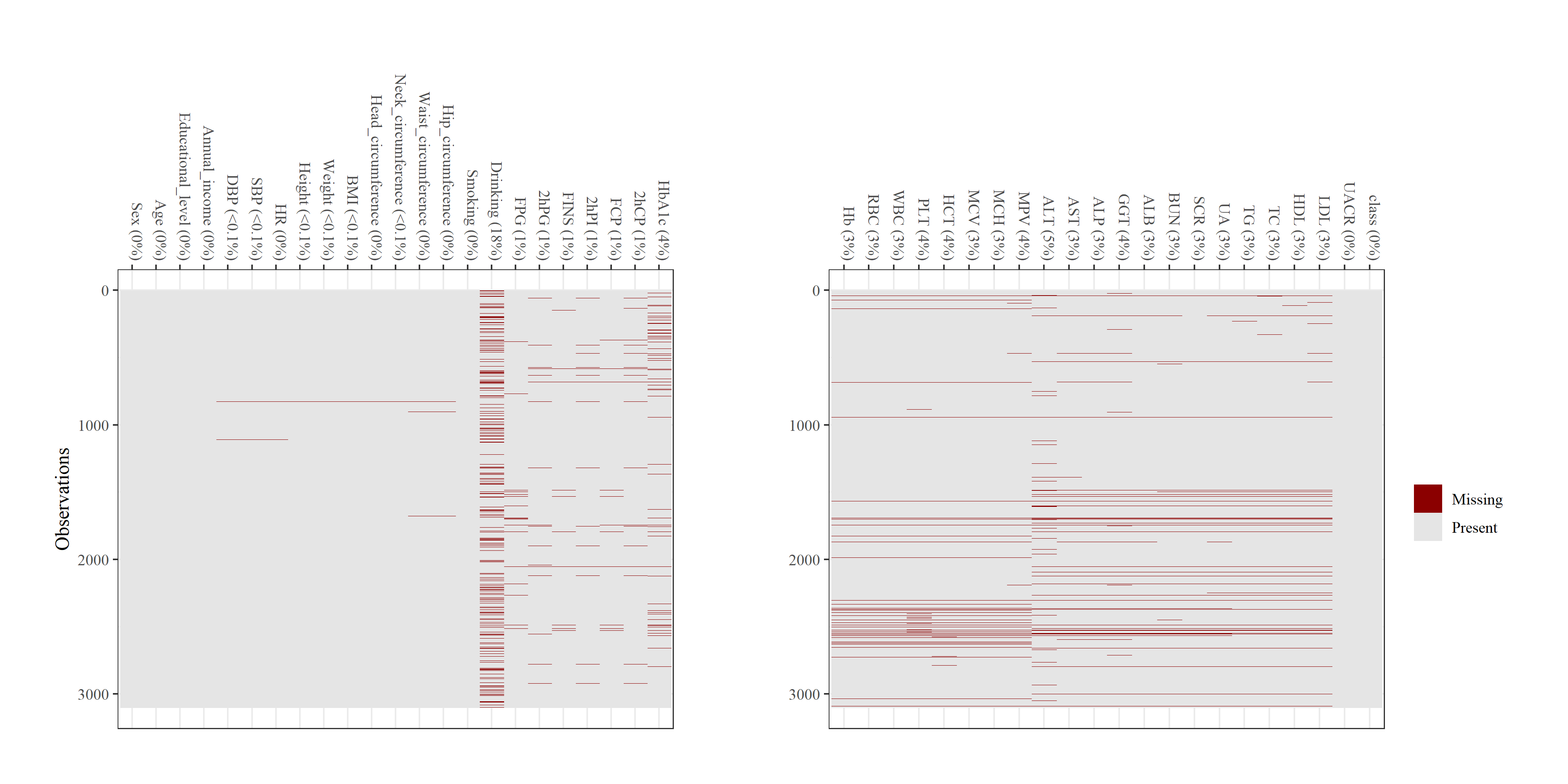


**Supplementary Figure 2. Proportion of Missing Values**

**Supplementary Table 1. Goodness-of-Fit Statistics for UACR Trajectories in T2DM Patients**

| Fit statistic | Number of classes | | | | |
| --- | --- | --- | --- | --- | --- |
|  | 1 | 2 | 3 | 4 | 5 |
| BIC | 90901.78 | 85683.82 | 84406.46 | 83949.28 | 83737.23 |
| AIC | 90877.62 | 85629.46 | 84321.90 | 83834.53 | 83592.29 |
| Class proportion | Class 1, 100% | Class 1, 85.55% | Class 1, 74.07% | Class 1, 68.85% | Class 1, 68.66% |
|  |  | Class 2, 14.45% | Class 2, 19.09% | Class 2, 19.09% | Class 2, 1.81% |
|  |  |  | Class 3, 6.84% | Class 3, 8.80% | Class 3, 19.57% |
|  |  |  |  | Class 4, 3.26% | Class 4, 7.22% |
|  |  |  |  |  | Class 5, 2.74% |
| average posterior probability (AvePP) |  | Class 1, 0.98 | Class 1, 0.95 | Class 1, 0.92 | Class 1, 0.92 |
|  |  | Class 2, 0.92 | Class 2, 0.82 | Class 2, 0.76 | Class 2, 0.85 |
|  |  |  | Class 3, 0.91 | Class 3, 0.81 | Class 3, 0.76 |
|  |  |  |  | Class 4, 0.89 | Class 4, 0.82 |
|  |  |  |  |  | Class 5, 0.91 |
